# Wood forming tissue‐specific bicistronic expression of *PdGA20ox1* and *PtrMYB221* improves both the quality and quantity of woody biomass production in a hybrid poplar

**DOI:** 10.1111/pbi.13036

**Published:** 2018-12-05

**Authors:** Jin‐Seong Cho, Hyung‐Woo Jeon, Min‐Ha Kim, The K. Vo, Jinsoo Kim, Eung‐Jun Park, Young‐Im Choi, Hyoshin Lee, Kyung‐Hwan Han, Jae‐Heung Ko

**Affiliations:** ^1^ Department of Plant & Environmental New Resources Kyung Hee University Yongin Korea; ^2^ Department of Chemical Engineering Kyung Hee University Yongin Korea; ^3^ Division of Forest Biotechnology Korea Forest Research Institute Suwon Korea; ^4^ Department of Horticulture and Department of Forestry Michigan State University East Lansing MI USA

**Keywords:** bicistronic gene expression, developing xylem promoter, *PdGA20ox1*, *PtrMYB221*, synthetic biology, poplar

## Abstract

With the exponential growth of the human population and industrial developments, research on renewable energy resources is required to alleviate environmental and economic impacts caused by the consumption of fossil fuels. In this study, we present a synthetic biological application of a wood forming tissue‐specific bicistronic gene expression system to improve both the quantity and quality of woody biomass to minimize undesirable growth penalties. Our transgenic poplars, designed to express both *PdGA20ox1* (a GA20‐oxidase from *Pinus densiflora* producing bioactive gibberellin, GA) and *PtrMYB221* (a MYB transcription factor negatively regulating lignin biosynthesis) under the developing xylem (DX) tissue‐specific promoter (i.e., *DX15::PdGA20ox1‐2A‐PtrMYB221* poplar), resulted in a 2‐fold increase in biomass quantity compared to wild‐type (WT), without undesirable growth defects. A similar phenotype was observed in transgenic Arabidopsis plants harboring the same gene constructs. These phenotypic consequences were further verified in the field experiments. Importantly, our transgenic poplars exhibited an improved quality of biomass with reduced lignin content (~16.0 wt%) but increased holocellulose content (~6.6 wt%). Furthermore, the saccharification efficiency of our transgenic poplar increased significantly by up to 8%. Our results demonstrate that the controlled production of both GA and a secondary wall modifying regulator in the same spatio‐temporal manner can be utilized as an efficient biotechnological tool for producing the desired multi‐purpose woody biomass.

## Introduction

Currently, a tremendous amount of fossil fuels is consumed to cope with the increasing demands of our modern society with exponential growth of population and industrial developments. The resulting environmental and economic impacts are shifting our research interests to various sources of renewable energy such as solar, wind, geothermal, tidal, and biomass (Blunden and Arndt, 2016; Fouquet, 2016). While most alternate energy sources produce electric energy, biomass can be converted into various forms of energy including not only electricity, but also gas and liquid energy, which can be used directly in current internal combustion engines (Albers *et al*., 2016; Chundawat *et al*., 2011; López‐Hidalgo *et al*., 2017; Vanholme *et al*., 2013). Recently, many studies have been conducted to produce biofuels from woody biomass, which account for more than 90% of the total biomass produced on Earth (Castro *et al*., 2014; Crawford *et al*., 2016; Ko *et al*., 2017; Serapiglia *et al*., 2013; Vo *et al*., 2017; Zhu *et al*., 2010). *Populus* species can serve as an ideal woody biomass because of their fast growth characteristics for feedstock supply and inherent secondary cell wall characteristics with suitable cellulose to lignin ratios for utilization as a bioethanol crop (Porth and El‐Kassaby, 2015).

Plant hormones play an essential role in the growth and development of plants. Among them, gibberellins (GAs) regulate many aspects of plant development, including stem elongation, flowering, and wood formation (Biemelt *et al*., 2004; Eriksson *et al*., 2000; Hedden and Thomas, 2012; Kende and Zeevaart, 1997; Kurosawa, 1926; Mauriat and Moritz, 2009; Park *et al*., 2015). It has been shown that GA 20‐oxidase (GA20ox) plays a key role in bioactive GA biosynthesis in several plant species (Carrera *et al*., 2000; Coles *et al*., 1999; Huang *et al*., 1998; Park *et al*., 2015; Rieu *et al*., 2008; Zi *et al*., 2014). Many studies have shown that overexpression of *GA20ox* resulted in the increase of stem elongation in Arabidopsis (Coles *et al*., 1999; Huang *et al*., 1998), potato (Carrera *et al*., 2000), hybrid aspen (Eriksson *et al*., 2000; Mauriat and Moritz, 2009), and cotton (Xiao *et al*., 2010). However, overproduction of GA by constitutive expression (i.e., using the CaMV 35S promoter) of *GA20ox* has been accompanied by undesirable pleiotropic side effects on plant growth, including poor rooting, small leaves, and slender stems (Eriksson *et al*., 2000; Jeon *et al*., 2016; Mauriat *et al*., 2014). Previously, we have reported a strategy that employed a production of GA using *PdGA20ox1*, which encodes GA20ox from *Pinus densiflora*, through a developing xylem (DX) tissue‐specific promoter (i.e., DX15 promoter; Ko *et al*., 2012) as an efficient biotechnological tool for producing enhanced plant biomass and reducing unwanted growth penalties (Jeon *et al*., 2016).

For an efficient bioenergy production using woody biomass, it is important to improve both the quantity and quality of wood. Secondary cell walls comprising woody biomass mainly consist of cellulose, hemicellulose, and lignin (Chundawat *et al*., 2011; Lee *et al*., 2011; Vanholme *et al*., 2013). To produce liquid biofuels (e.g., bioethanol), cellulose and hemicellulose are utilized as fermentable sugars (Chundawat *et al*., 2011; Vanholme *et al*., 2013). However, lignins, which are complex polymers of phenolic compounds, contribute to the recalcitrance of the secondary walls to deconstruction because of tight cross‐links with cellulose and hemicellulose fibrils (Carroll and Somerville, 2009; Pauly and Keegstra, 2010; Simmons *et al*., 2010; Somerville *et al*., 2010). Therefore, adjusting the amount and ratio of the secondary wall components is very important in enhancing the production and yield of biofuels (Albers *et al*., 2016; Chundawat *et al*., 2011; Vanholme *et al*., 2013).

Several MYB transcription factors (TF) have been identified as major regulators of secondary wall formation, and MYB46 plays a pivotal role as a master switch for secondary wall biosynthesis in Arabidopsis (Kim *et al*., 2013; Ko *et al*., 2009, 2014; McCarthy *et al*., 2009). However, some MYB TFs have been reported as inhibitors of lignin biosynthesis (Raes *et al*., 2003; Zhao and Dixon, 2011); for example, overexpression of eucalyptus *EgMYB1*, corn *ZmMYB31*, or switchgrass *PvMYB4* decreases lignin content (Fornalé *et al*., 2006; Legay *et al*., 2010; Shen *et al*., 2012; Sonbol *et al*., 2009). Recently, poplar MYB TFs similar to EgMYB1, such as PtoMYB156 and PdMYB221, were also found to be involved in negative regulation of lignin biosynthesis as well as secondary wall formation (Tang *et al*., 2015; Yang *et al*., 2017).

A multi‐cistronic gene expression system was successfully utilized in both plants and animals to express two or more transgenes in synchronized coordinated expression under the control of a single promoter (Daniels *et al*., 2014; Ha *et al*., 2010; Lee *et al*., 2012; Mikkelsen *et al*., 2010; Tian *et al*., 2013; Trichas *et al*., 2008; Zhang *et al*., 2017). This system encodes multiple genes in a single open reading frame, with a short intervening viral 2A sequence, which has self‐processing properties, between two coding sequences (Atkins *et al*., 2007; Donnelly *et al*., 2001). The 2A self‐cleaving peptide (2A), identified from foot‐and‐mouth‐disease virus (FMDV), is an oligopeptide (usually 19–22 amino acids) located between two proteins (Ryan *et al*., 1991). The 2A peptide self‐cleaves to generate two proteins by a translational effect known as ‘stop‐go’ or ‘stop‐carry’ (Atkins *et al*., 2007; Donnelly *et al*., 2001; Ryan and Drew, 1994). Because the 2A system has many advantages, it has been widely applied in many areas of research including gene therapy, gene function study, and production of genetically modified organisms (Daniels *et al*., 2014; Ha *et al*., 2010; Lee *et al*., 2012; Mikkelsen *et al*., 2010; Tian *et al*., 2013; Trichas *et al*., 2008; Zhang *et al*., 2017).

In this study, we took advantage of the viral 2A system in a synthetic biological approach to express two genes in developing xylem tissue specifically to improve woody biomass. Our results showed that the controlled production of GA and a transcriptional regulator for secondary wall biosynthesis through a DX15 promoter successfully produced improved woody biomass both quantitatively and qualitatively without any growth penalties.

## Results

### Strategy to generate transgenic plants expressing both *PdGA20ox1* and *PtrMYB221* in developing xylem tissue

To improve both quantity and quality of woody biomass while minimizing undesirable growth penalties, we applied two biotechnological tools in this study: One is the ‘viral 2A self‐cleaving peptide’ to express two genes (*PdGA20ox1* and *PtrMYB221*) bicistronically (Atkins *et al*., 2007; Donnelly *et al*., 2001; Mikkelsen *et al*., 2010; Ryan and Drew, 1994) (Figure S1); the other is the ‘DX15 promoter’ for DX tissue‐specific expression (Ko *et al*., 2012). *PdGA20ox1*, a GA20ox1 from *P. densiflora*, was used to increase biomass ‘quantity’ as we reported (Jeon *et al*., 2016; Park *et al*., 2015), while *PtrMYB221*, a MYB TF from *Populus trichocarpa*, was used to improve biomass ‘quality’ because *PtrMYB221* is the closest homolog of *EgMYB1* (Figure S2), a well‐known negative regulator of lignin biosynthesis (Legay *et al*., 2010). The ‘quality’ in this study indicates the changes of wood property (e.g., reduction of lignin contents) to improve saccharification efficiency.

Thus, this system allows the expression of two genes that affect both the quantity and quality of woody biomass production in the same spatio‐temporal manner in developing xylem tissue (Figure S1). Using this system, we generated both transgenic Arabidopsis and poplar plants, designated *DX15::PdGA20ox1‐2A‐PtrMYB221* plants.

### Transgenic Arabidopsis plants showed increased biomass formation

**Figure 1:**
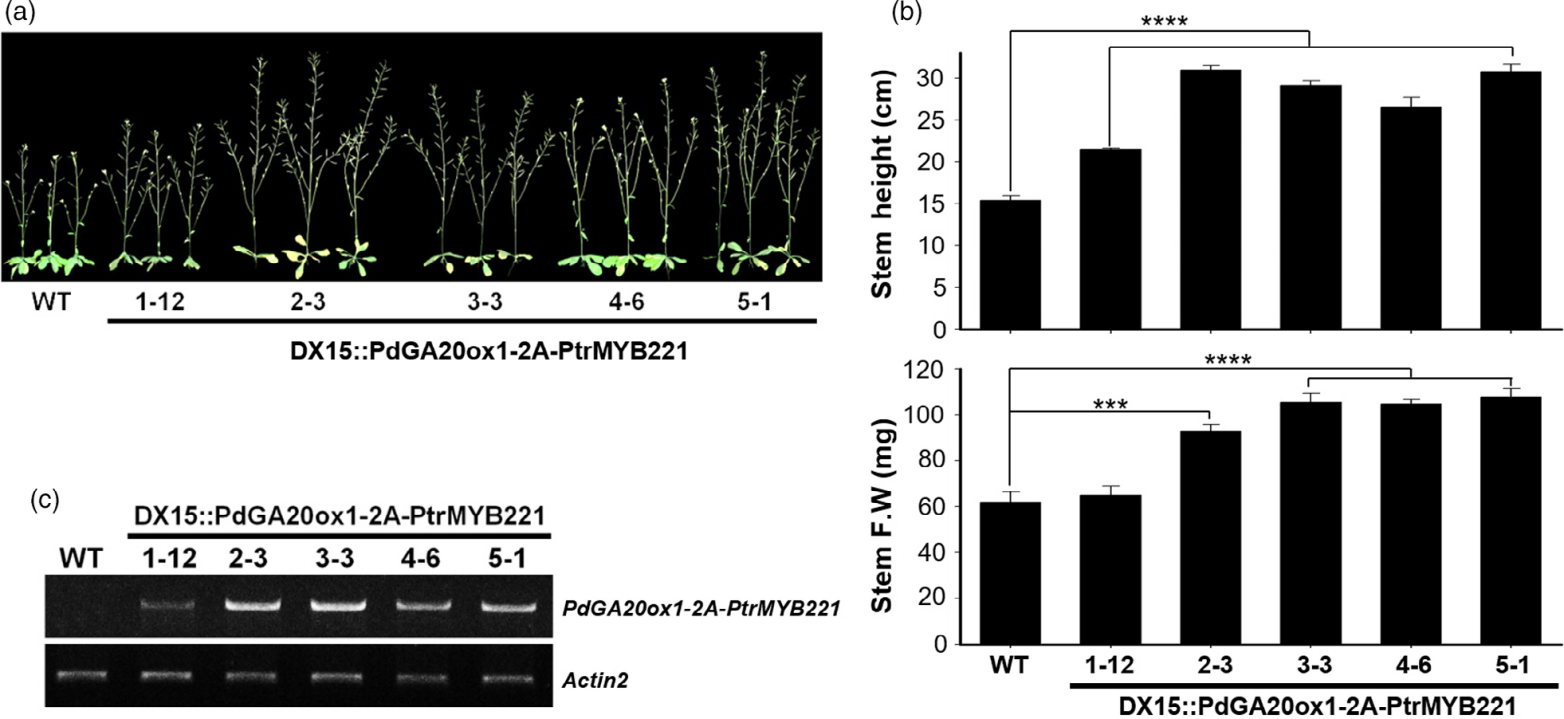
Enhanced growth of transgenic Arabidopsis plants. Growth performances were analyzed using 43‐day‐old soil‐grown plants. (a) Overall growth phenotypes of transgenic Arabidopsis plants (i.e., DX15::PdGA20ox1‐2A‐PtrMYB221) were compared to those of wild‐type (WT) plants. Five independent T3 homozygote transgenic lines (e.g., 1‐12, 2‐3, 3‐3, 4‐6, and 5‐1) are shown. (b) Biomass increases of transgenic Arabidopsis plants compared to WT plants. Both stem height (upper) and stem fresh weight (lower) were measured. Error bars indicate SE (n = 6) (***P‐value < 0.001, ****P‐value <0.0001, unpaired *t*‐test). (c) Expression of PdGA20ox1‐2A‐PtrMYB221 transcripts in transgenic Arabidopsis plants. Semi‐quantitative RT‐PCR (26 cycles) was performed using cDNA templates generated from stem total RNA. PdGA20ox1 forward primer (#5) and PtrMYB221 reverse primer (#6) were used to amplify the *PdGA20ox1‐2A‐PtrMYB221* transcripts (Table S1). The Actin2 gene was used as a loading control.

For a proof‐of‐concept experiment, we performed phenotypic analysis of transgenic Arabidopsis plants (i.e., *DX15::PdGA20ox1‐2A‐PtrMYB221*) using five selected T3 homozygous lines (Figure 1). We observed significant increases of biomass in 43‐day‐old soil‐grown transgenic Arabidopsis plants compared to WT plants (Figure 1), which is likely caused by an elevated endogenous GA level in DX tissue and is consistent with our previous observation of transgenic Arabidopsis with a *DX15::PdGA20ox1* construct (Jeon *et al*., 2016). Compared to WT plants, the transgenic Arabidopsis had a much taller stem height by up to 2‐fold, and the fresh weights of the stem increased by up to 1.88‐fold (Figure 1a,b). These phenotypic consequences of transgenic Arabidopsis are in line with the expression level of the introduced gene, a *PdGA20ox1‐2A‐PtrMYB221* transcripts (Figure 1c).

### Transgenic poplar trees resulted in an increase of biomass

**Figure 2:**
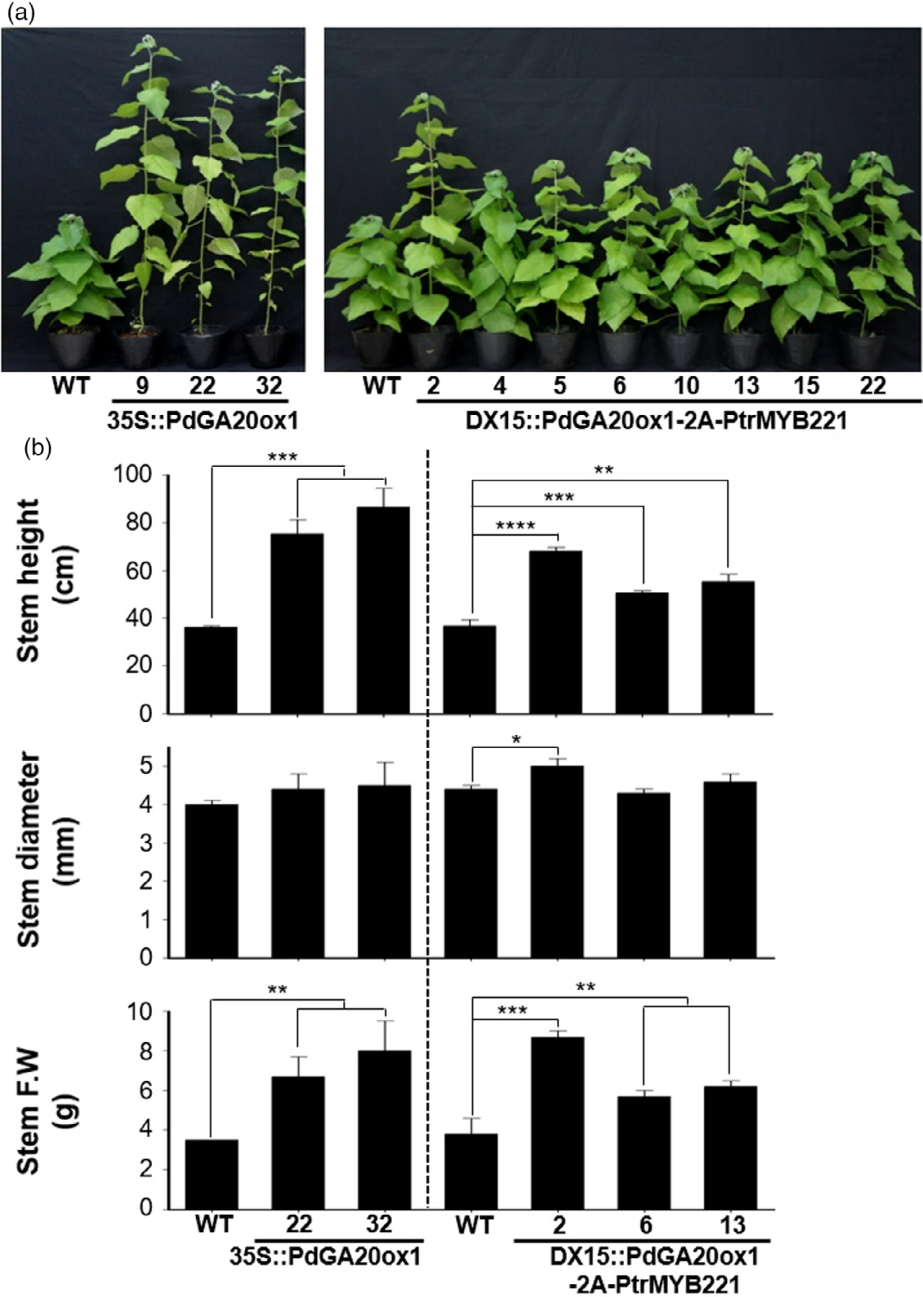
Biomass increases of transgenic poplar plants grown in a greenhouse. Growth performances were analyzed using 60‐day‐old soil‐grown poplar plants. (a) Overall growth phenotype of transgenic poplar plants (DX15::PdGA20ox1‐2A‐PtrMYB221) compared to wild‐type (WT) and 35S::PdGA20ox1 poplar plants. Numbers in each transgenic poplar plant indicate independent lines. (b) Biomass increases of transgenic poplar plants (DX15::PdGA20ox1‐2A‐PtrMYB221) compared to WT and 35S::PdGA20ox1 poplar plants. Stem height (top), stem diameter (middle), and stem fresh weight (bottom) were measured from representative lines of each transgenic poplar plant. Error bars indicate SD (n = 5) (*P‐value < 0.1, **P‐value < 0.01, ***P‐value < 0.001, ****P‐value <0.0001, unpaired *t*‐test).

As expected from our previous results (Jeon *et al*., 2016; Park *et al*., 2015), *DX15::PdGA20ox1‐2A‐PtrMYB221* transgenic poplars showed enhanced biomass production comparable to that of *35S::PdGA20ox1* poplars (Figure 2). We analyzed the overall growth of young plants grown in a greenhouse for 60 days. The height of the stems was increased by up to 2.39 and 1.85‐fold in *35S::PdGA20ox1* and *DX15::PdGA20ox1‐2A‐PtrMYB221,* respectively, compared to WT (Figure 2b). In addition, stem fresh weights of both transgenic poplars were increased by up to 2.29‐fold compared to WT (Figure 2b).

Although the DX‐specific expression capacity of the DX15 promoter has been confirmed (Ko *et al*., 2012), we verified stem‐specific expression of *PdGA20ox1* in *DX15::PdGA20ox1‐2A‐PtrMYB221* transgenic poplar plants by both semi‐quantitative RT‐PCR and quantitative RT‐PCR (Figure S3). While the *PdGA20ox1* gene in *35S::PdGA20ox1* transgenic poplar was strongly expressed in both stems and leaves, the *PdGA20ox1* gene in *DX15::PdGA20ox1‐2A‐PtrMYB221* transgenic poplar was specifically expressed in stem tissue only, although the overall expression level was lower than that of *35S::PdGA20ox1* transgenic poplar (Figure S2).

**Figure 3:**
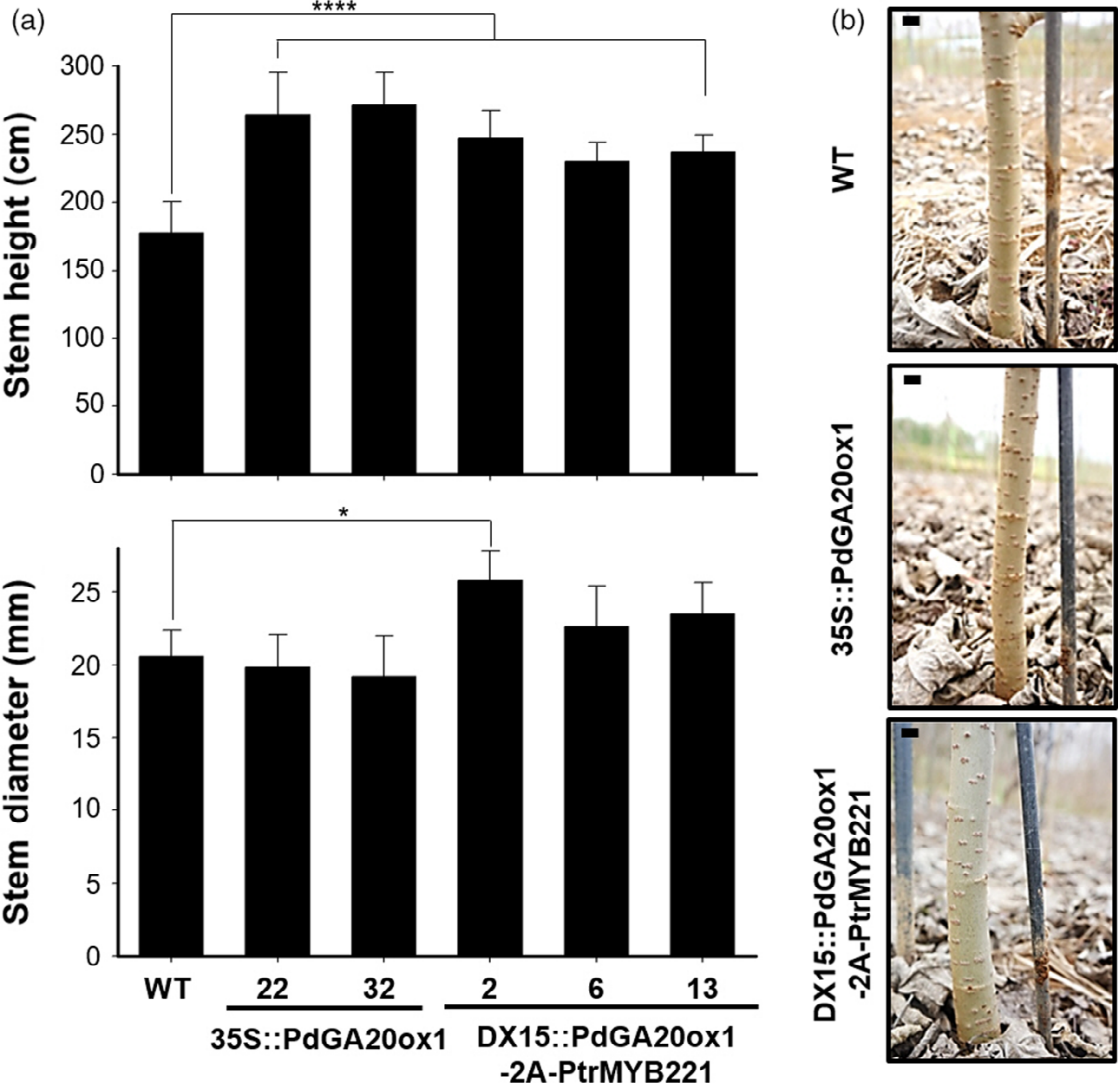
Biomass increases of transgenic poplar plants grown in an LMO field. (a) Growth performances were analyzed using poplar plants grown in an LMO field for 3 months (from spring to summer). Stem height (upper) and stem diameter (lower) were measured in transgenic poplar plants (DX15::PdGA20ox1‐2A‐PtrMYB221) compared to wild‐type (WT) and 35S::PdGA20ox1 poplar plants. Error bars indicate SD (n = 5) (*P‐value <0.1, ****P‐value <0.0001, unpaired *t*‐test). (b) Stem diameter growth of DX15::PdGA20ox1‐2A‐PtrMYB221 transgenic poplar plant (#2) compared to 35S::PdGA20ox1 (#32) and WT plants grown for 6 months in an LMO field. Representative pictures are shown. Scale bars indicate 1 cm.

To assess biomass formation in field conditions, we measured the height and stem diameter of transgenic poplars grown for 3 months (i.e., active growing season from spring to summer) in a living modified organism (LMO) site (Figure 3). Height was increased up to 1.53‐fold and 1.39‐fold in *35S::PdGA20ox1* and *DX15::PdGA20ox1‐2A‐PtrMYB221* poplars, respectively, compared to WT. Interestingly, stem diameter was increased by up to 1.25‐fold in *DX15::PdGA20ox1‐2A‐PtrMYB221* poplars, while no significant differences were found in *35S::PdGA20ox1* poplars compared to WT (Figure 3a). After 6 months of growth, the stem diameter was greater in the *DX15::PdGA20ox1‐2A‐PtrMYB221* poplars compared to both WT and *35S::PdGA20ox1* plants (Figure 3b).

### Undesirable growth penalties caused by GA overproduction were minimized in the *DX15::PdGA20ox1‐2A‐PtrMYB221* transgenic poplar

**Figure 4:**
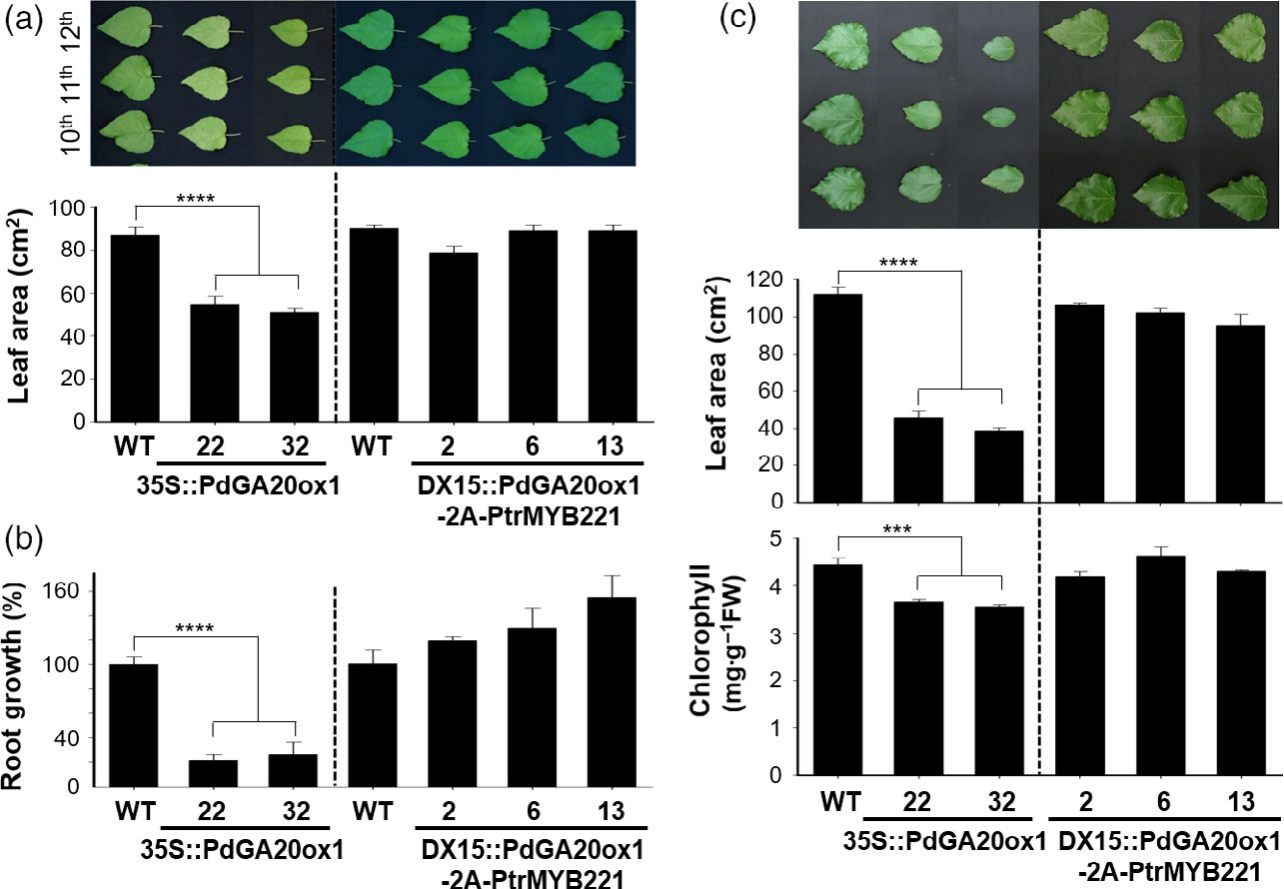
Undesirable growth penalties were minimized in the DX15::PdGA20ox1‐2A‐PtrMYB221 transgenic poplar. (a and b) Leaf and root growths were analyzed using 60‐day‐old greenhouse‐grown poplar plants. Leaf area is the average of three of the 10th–12th leaves from the top. Representative leaf pictures are shown (top of (a)). Root fresh weights were measured for root growth (b). (c) Leaf growth (middle) and chlorophyll content (bottom) were analyzed using poplar plants grown in the LMO field for 3 months (from spring to summer). Both leaf area and chlorophyll content are the average value of three leaves, which are the largest of the 10th–18th leaves from the top. Representative leaf pictures are shown (top). Error bars indicate SE (n = 5) (***P‐value < 0.001, ****P‐value <0.0001, unpaired *t*‐test).

Jeon *et al*. (2016) reported a successful utilization of the DX15 promoter for controlled GA production to reduce undesirable growth penalties (e.g., weak stems, poor root growth, and leaf developments) reported in GA overproducing poplar plants by overexpressing *GA20ox* (Eriksson *et al*., 2000; Mauriat *et al*., 2014). Consistent with our previous results (Jeon *et al*., 2016), *35S::PdGA20ox1* poplars (lines 22 and 32) grown for 60 days in a greenhouse exhibit a reduction in leaf area of approximately 38%–40%, and leaves are a pale green color compared to the WT (Figure 4a). However, the leaf growth of *DX15::PdGA20ox1‐2A‐PtrMYB221* poplars is quite comparable to that of WT (Figure 4a). Root growth, critical for sustainable growth of plants, was severely affected in *35S::PdGA20ox1* poplars, as reported previously. Figure 4b shows that *35S::PdGA20ox1* lines had a reduction of root fresh weight of around 73%–78% compared to WT. However, *DX15::PdGA20ox1‐2A‐PtrMYB221* lines showed similar or better growth of roots than did the WT (Figure 4b).

In LMO field conditions, leaf growth of *35S::PdGA20ox1* poplars was severely reduced by up to 65%, whereas that of *DX15::PdGA20ox1‐2A‐PtrMYB221* line 2 showed no changes compared to WT (Figure 4c). The chlorophyll content of leaves was measured to estimate photosynthetic activity. Although there was a significant reduction of chlorophyll content in the leaves of *35S::PdGA20ox1* poplars, *DX15::PdGA20ox1‐2A‐PtrMYB221* lines exhibited no differences compared to WT (Figure 4c).

Taken together, these results indicate that the preferential expression of wood forming tissue of both *PdGA20ox1* and *PtrMYB221* substantially minimized undesirable phenotypes compared to the *35S::PdGA20ox1* poplar.

### Transgenic poplars produce an improved quality of woody biomass

**Figure 5:**
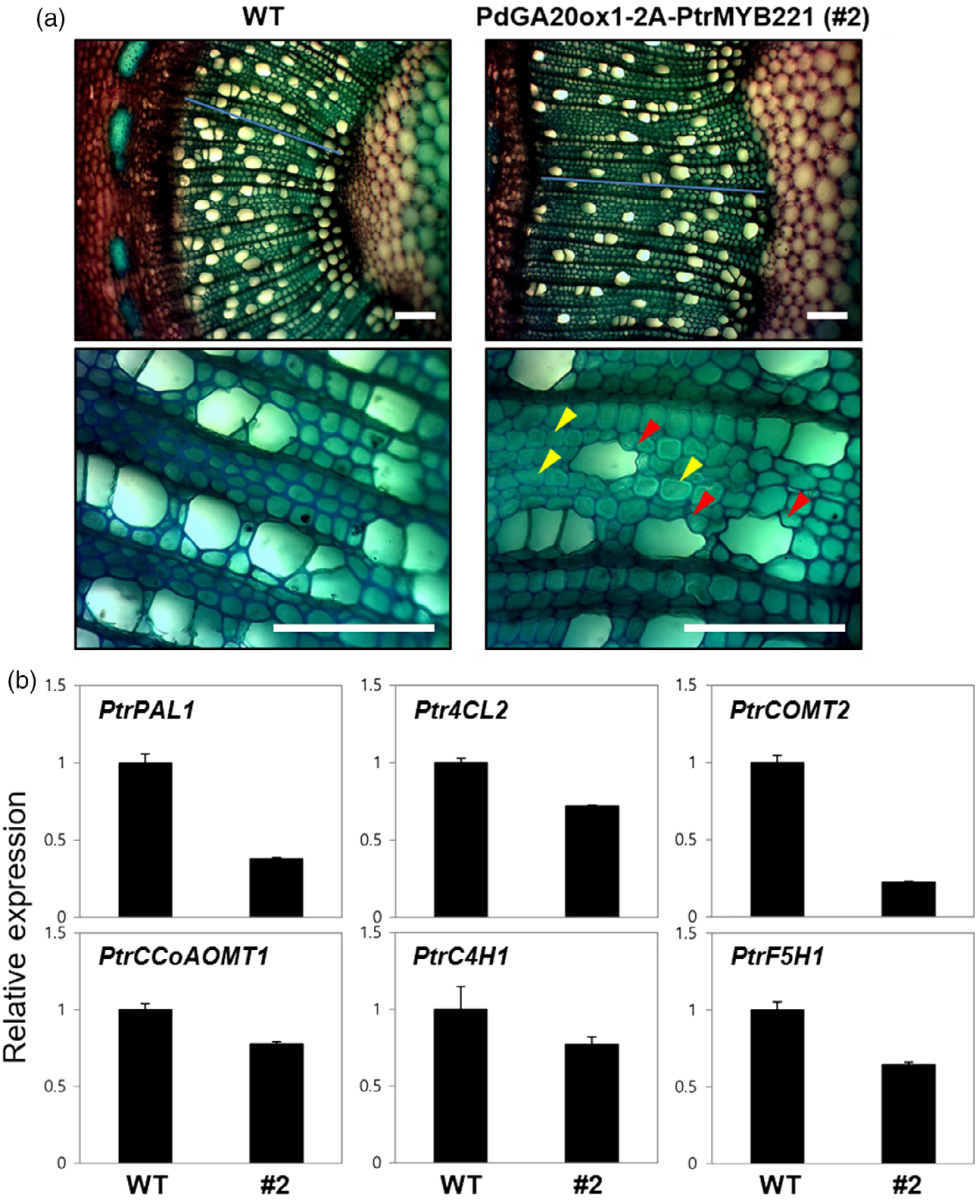
Transgenic poplars produce improved quality of woody biomass. (a) Enhanced wood formation but reduced secondary wall thickening in DX15::PdGA20ox1‐2A‐PtrMYB221 transgenic poplars. The 15th internodes of the main stems from 60‐day‐old soil‐grown poplar plants were used for histological analysis. Transverse stem sections were stained with toluidine blue *O*. Lower panels are the enlarged view of upper panels. Red arrowheads indicate irregular xylem, and yellow arrowheads point to gelatinous fiber formation. Scale bars indicate 100 μm. (b) Expression of genes involved in the lignin biosynthetic pathway. PtrPAL1 (phenylalanine ammonia‐lyase1, Potri.006G126800.1), Ptr4CL2 (4‐coumaroyl‐CoA ligase2, Potri.019G049500.2), PtrCOMT2 (caffeic acid 3‐O‐methyltransferase2, Potri.012G006400.2), PtrCCoAOMT1 (caffeoyl‐CoA 3‐O‐methyltransferase1, Potri.001G304800.1), PtrC4H1 (cinnamate 4‐hydroxylase1, Potri.013G157900.1), and PtrF5H1 (ferulate 5‐hydroxylase1, Potri.007G016400.1). Quantitative real‐time PCRs were performed using first‐strand cDNA synthesized from total RNAs extracted from stem tissues of 60‐day‐old WT plants and the #2 line of the DX15::PdGA20ox1‐2A‐PtrMYB221 poplar. Relative transcript levels were determined using the PtrACTIN genes as a quantitative control. Error bars indicate SD of three independent experiments.

We examined the stem tissues of the *DX15::PdGA20ox1‐2A‐PtrMYB221* transgenic poplars to observe the quantitative and qualitative changes of wood formation. A stem cross section at the 15th internode from the 60‐day, greenhouse‐grown poplars showed that, compared to WT, all the transgenic lines exhibited a substantial increase in secondary xylem differentiation with gelatinous fiber formation (Figure 5a), which is consistent with our previous transgenic poplars expressing either *35S::PdGA20ox1* or *DX15::PdGA20ox1* (Jeon *et al*., 2016; Park *et al*., 2015). Very interestingly, our *DX15::PdGA20ox1‐2A‐PtrMYB221* poplars showed an irregular xylem phenotype that has never been observed (Figure 5a). This result suggests that *DX15::PdGA20ox1‐2A‐PtrMYB221* poplars have reduced secondary wall thickening in the xylem, most likely caused by reduced lignin content through PtrMYB221 function. This phenotype was consistent to the transgenic Arabidopsis plants in our proof‐of‐concept experiment (Figure 1). Among five selected T3 homozygous lines, three lines (e.g., #2‐3, #3‐3, #4‐6) showed clear irregular xylem phenotype (Figure S4) and these phenotypic consequences seem to closely associate with the expression level of the introduced *PdGA20ox1‐2A‐PtrMYB221* transcripts (Figure 1 and Figure S4).

Accordingly, the expression of many of the lignin biosynthetic genes, such as *PtrPAL1*,* Ptr4CL2*,* PtrCOMT2*,* PtrCCOAOMT1*,* PtrC4H1*, and *PtrF5H1*, was significantly reduced (Figure 5b). Indeed, *DX15::PdGA20ox1‐2A‐PtrMYB221* poplars have increased cellulose but substantially decreased lignin content (~16 wt%) in wood tissues compared to those of WT (Figure S5), which was reported previously (Vo *et al*., 2017).

**Figure 6:**
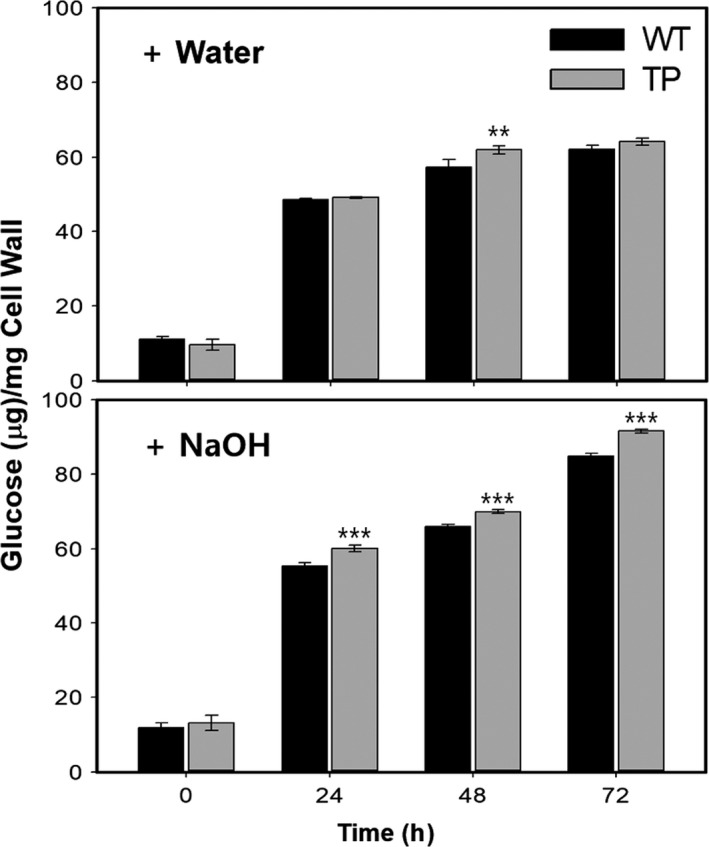
Increased saccharification efficiency of cell wall materials from transgenic poplar plants. Saccharification efficiency was estimated by analyzing the glucose content produced by cell wall materials of woody stems of poplar plants grown in the LMO field for 3 months (from spring to summer) after water (upper) or NaOH (lower) treatment for the indicated time (see Methods). WT, wild type; TP, transgenic poplar plants (DX15::PdGA20ox1‐2A‐PtrMYB221). Error bars indicate SD (n = 9) (**P‐value < 0.01, ***P‐value <0.001, unpaired *t*‐test).

Saccharification efficiency of wood materials from LMO field grown poplars was estimated by quantifying the amount of glucose released at different incubation times after hot water or alkali (NaOH) pretreatment (Figure 6). Our results showed that a significant increase in saccharification efficiency was observed in NaOH treated transgenic poplars compared to WT, up to 8% at 24 h (Figure 6). However, no significant changes were found in hot water treatment except after incubation for 48 h. These results suggest that our transgenic poplars have enhanced wood formation but reduced lignin content, which probably confers improved saccharification efficiency.

## Discussion

Previously, we reported that a *GA20ox1* gene from *P. densiflora* (i.e., *PdGA20ox1*) and overexpression of *PdGA20ox1* in a hybrid poplar resulted in an elevated level of endogenous GA and enhanced stem growth and wood formation (Park *et al*., 2015). However, transgenic poplars constitutively overexpressing *PdGA20ox1* (i.e., *35S::PdGA20ox1* poplar) showed unwanted phenotypes such as poor rooting and small leaves with weak stems, as reported previously (Eriksson *et al*., 2000; Mauriat *et al*., 2014). To reduce undesirable phenotypes of the *35S::PdGA20ox1* poplar, we utilized the DX15 promoter and produced *DX15::PdGA20ox1* poplars (Jeon *et al*., 2016). As expected, the biomass production of *DX15::PdGA20ox1* poplars was comparable to the *35S::PdGA20ox1* poplar but with significantly reduced growth defects (Jeon *et al*., 2016).

Here, we describe a one‐step advanced strategy aimed at improving both the quantity and quality of woody biomass while minimizing the undesirable growth penalties by expressing *PdGA20ox1* and secondary wall modifying TFs in the same spatio‐temporal manner. We tested a *PtrMYB221* gene (Potri.004G174400.1) as a secondary wall modifying TF to reduce lignin content in wood tissue. The *PtrMYB221* gene is predicted to function as a transcriptional repressor with a DLNLEL motif in the C‐terminal region (Figure S2). DLNLEL is one of the ERF‐associated amphiphilic repression (EAR) motifs that is conserved in transcriptional repressors including *EgMYB1*,* ZmMYB31*, and *PvMYB4* (Fornalé *et al*., 2006; Legay *et al*., 2010; Ohta *et al*., 2001; Shen *et al*., 2012; Sonbol *et al*., 2009). Overexpressing *EgMYB1* in transgenic poplar decreases the lignin content, indicating that EgMYB1 acts as the negative regulator of *PAL2, C4H2, C3H1, F5H, CCoAOMT1, CCR2*, and *CAD1* genes (Legay *et al*., 2010). In addition, the *PtrMYB221* gene is orthologous to *PdMYB221* from *Populus deltoides* and *PtoMYB156* from *Populus tormentosa*, which were recently identified as repressors of both phenylpropanoid biosynthesis and secondary cell wall formation in poplar (Tang *et al*., 2015; Yang *et al*., 2017).

Our *DX15::PdGA20ox1‐2A‐PtrMYB221* transgenic poplars resulted in an improved biomass quantity comparable to those of *35S::PdGA20ox1* and *DX15::PdGA20ox1* poplars, without any growth defects. For examples, the fresh weights of stems from transgenic poplars that were greenhouse‐grown for 60 days were increased by up to 2.29‐fold (Figure 2), and the height and stem diameter of three‐month transgenic poplars grown in LMO fields increased by up to 1.39‐ and 1.25‐fold, respectively, compared to WT (Figure 3). Thus, biomass (calculated with stem height and width) was increased by around 2.17‐fold in LMO field‐grown transgenic poplars. This quantitative increase of biomass is most likely from the wood forming tissue‐preferential production of GA by *PdGA20ox1*, as we have reported (Jeon *et al*., 2016; Park *et al*., 2015). However, root and leaf have no signs of growth defects in transgenic poplars (Figure 4). We speculate that this might come from an adequate increase of GA level in wood forming tissue specifically due to reduced *PdGA20ox1‐2A‐PtrMYB221* transcripts in our *DX15::PdGA20ox1‐2A‐PtrMYB221* transgenic poplars compared to *35S::PdGA20ox1* (Figure S3).

Importantly, our transgenic poplars exhibited an improved quality of biomass. The lignin content of woody stems decreased by around 16.0 wt%, while holocellulose (cellulose + hemicellulose) content was increased by up to 6.6 wt% (Figure S5). Indeed, transgenic poplars revealed an irregular xylem phenotype due to reduced secondary wall thickening, most likely caused by decreased lignin biosynthesis (Figure 5). In fact, most of the lignin biosynthetic genes were decreased (Figure 5a), suggesting that PtrMYB221 functions as a negative regulator of lignin biosynthesis. However, secondary xylem formation (i.e., width of secondary xylem) was increased more than 45% compared to WT (Figure 5a), and gelatinous fibers, which are cellulose‐rich, were clearly developed (Clair *et al*., 2006). These results are consistent with those from our previous transgenic poplars (Jeon *et al*., 2016; Park *et al*., 2015) and may explain the dramatic increase of cellulose content (~28 wt%) in *DX15::PdGA20ox1‐2A‐PtrMYB221* transgenic poplars compared to WT poplars (Figure S4). A similar observation reported that the repression of lignin biosynthesis promotes cellulose accumulation in transgenic poplars (Hu *et al*., 1999).

It has been widely accepted that an increase in hollocellulose content and a decrease in lignin content are beneficial to bio‐oil production via pyrolysis (Hu *et al*., 2016; Kan *et al*., 2016). Our previous study of pyrolysis characteristics and kinetics showed that transgenic poplars required a lower input energy for thermal decomposition and yielded a higher quantity of bio‐oil than the WT under the same pyrolysis conditions in isothermal pyrolysis experiments (Vo *et al*., 2017). Furthermore, saccharification efficiency in our transgenic poplar increased significantly by up to 8% at 24 h (Figure 6). Lignin imparts waterproofness, durability, and mechanical strength to the secondary cell wall. These properties of lignin act as a serious inhibitor during the saccharification process of woody materials (i.e., secondary xylem) for liquid biofuel production (Carroll and Somerville, 2009; Simmons *et al*., 2010). Thus, the increased saccharification efficiency in our transgenic poplars suggests that ligation of the secondary cell walls might have been loosened to decrease mechanical strength due to reduced lignin biosynthesis.

Interestingly, the reduced lignin contents in the transgenic poplars seem to not affect the growth fitness in the field condition. Our over‐winter growth analysis in the LMO field showed that all the DX15::PdGA20ox1‐2A‐PtrMYB221 plants were survived after winter season, while 35S::PdGA20ox1 poplars showed only 20%–40% of survival rate (Figure S6a), possibly by the precocious bud flushing (Figure S6b) due to high level of GA contents in winter buds. These results suggest that our DX15::PdGA20ox1‐2A‐PtrMYB221 plants are no different from WT poplars in surviving winter, a period of extensive abiotic stresses including cold, freezing and drought.

Although a multi‐cistronic gene expression system with the 2A self‐cleaving peptide (2A) has been successfully utilized in both plants and animals (Daniels *et al*., 2014; Ha *et al*., 2010; Lee *et al*., 2012; Mikkelsen *et al*., 2010; Tian *et al*., 2013; Trichas *et al*., 2008; Zhang *et al*., 2017), no report has yet been made in *Populus* tree species. Our transgenic poplars exhibiting both improved quantity and quality of woody biomass suggest that the 2A system may be operated to produce PdGA20ox1 and PtrMYB221 proteins, respectively. However, a detailed molecular analysis will be necessary to confirm the production of these proteins.

In summary, we utilized the viral 2A system in a synthetic biological approach to express both *PdGA20ox1* and *PtrMYB221* in wood forming‐tissue specifically to improve woody biomass. The resulting transgenic poplars showed a superior woody biomass without growth penalties. Thus, by substituting suitable transcriptional regulators as demonstrated (Ko *et al*., 2017), our strategy can be utilized as an efficient biotechnological tool for producing the desired woody biomass and expanded to various bioenergy crops.

## Experimental procedures

### Plant materials and growth conditions

*Arabidopsis thaliana*, ecotype Columbia (Col‐0), was used in both wild‐type and transgenic plant experiments. Arabidopsis were grown in soil in a growth room (14 h light; light intensity, 150 μmol/m^2^/s) at 23 °C or on half‐strength Murashige and Skoog medium (MS, Sigma‐Aldrich) containing 2% sucrose with appropriate antibiotics for screening. Hybrid poplars (*Populus alba* × *P. tremular* var. *glandulosa*, clone BH) were used as both wild‐type controls and transgenic plants in this study. The plants were acclimated in soil and grown in controlled conditions in a growth room (16 h light; light intensity, 150 μmol/m^2^/s; 24 °C).

### Vector construction and plant transformation

The full‐length cDNAs encoding *PdGA20ox1* and *PtrMYB221* were amplified by polymerase chain reaction (PCR) from cDNA of *P. densiflora* and *P. trichocarpa*, respectively. A virus‐derived 2A peptide sequence was used to produce a fusion gene of *PdGA20ox1* (without the stop codon) and *PtrMYB221* expressed with the DX15 promoter (Figure S1). We modified the 2A nucleotide sequence consisting of 13 amino acids to be optimized for codon usage of poplar, and the fusion gene was inserted downstream of the DX15 promoter in the DX15‐pMDC32 vector (Jeon *et al*., 2016) using the Gateway cloning system. The vector constructs were then introduced into *Agrobacterium tumefaciens* strain C58, which was used to transform Arabidopsis and poplar by the floral‐dip method (Clough and Bent, 1998) and leaf disk transformation‐regeneration method (Choi *et al*., 2005; Horsch *et al*., 1985), respectively. All of the constructs used in this study were verified by DNA sequencing.

### Histological analysis

Poplar main stems (15th internode) from 60‐day‐old soil‐grown plants or rosette level stem of Arabidopsis plants were used to obtain hand‐cut cross‐sections and stained with either 0.05% toluidine blue *O* or 2% phloroglucinol/HCl for 1 min as described previously (Jeon *et al*., 2016).

### RNA extraction and RT‐PCR

Total RNAs of Arabidopsis were extracted using Trizol reagent (Life Technologies, Carlsbad, CA) as described previously (Jeon *et al*., 2016). Total RNAs of poplar were extracted using the cetyltrimethylammonium bromide (CTAB) method with slight modification (Logemann *et al*., 1987). In brief, a fine powder of plant tissues was mixed with CTAB buffer, followed by phenol:chloroform:isoamyl alcohol (25:24:1) extraction. Isopropanol was added to the mixture to isolate RNA. One microgram of total RNA was reverse‐transcribed using Superscript III reverse transcriptase (Invitrogen, Carlsbad, CA) in 20 μL reactions. Subsequent RT‐PCR was performed with 1 μL of the reaction product as a template. Quantitative real‐time PCR was performed using the CFX96^TM^ Real‐Time PCR Detection System (Bio‐Rad, Hercules, CA) with iQ^TM^ SYBR^®^ Supermix (Bio‐Rad). Poplar *ACTIN2* gene was used as the internal quantitative control, and relative expression level was calculated by the 2−▵▵Ct method (Pfaffl, 2001). All primer sequences were designed using Primer3 software (http://fokker.wi.mit.edu). Sequences are provided in Table S1.

### Measurement of growth parameters of poplar

Overall growth parameters of 2‐month‐old, greenhouse‐grown poplars transplanted and grown for 3 months at LMO sites (latitude 37.2N, longitude 126.9E) were measured. Parameters assessed were stem height (measured from top to bottom) and diameter (stem thickness measured at 10 cm above the soil level using slide calipers). Three leaves, the largest from the 10th to18th leaves from the top, were used for measurements of leaf area and chlorophyll content with an LI‐3100 area meter (LI‐COR Biosciences, Lincoln, NE) and ethanol extraction method (Lichtenthaler, 1987), respectively.

### Saccharification efficiency of transgenic poplar

Saccharification efficiency of transgenic poplars grown for 3 months at LMO sites was measured. Stem tissues were dried at 65 °C for 3 days and ground to a fine powder. Reducing sugar content was determined using the method of Yang *et al*. (2013) with slight modification. Briefly, for pretreatment, the ground materials (~2 mg) were transferred into a 2‐mL screw‐cap tube and incubated with 200 μL of water or 180 μL of NaOH (1%, w/v) at 30 °C for 30 min and autoclaved at 120 °C for 60 min. After cooling to room temperature, 200 μL of water was added to water treated sample, while 200 μL of 2.5 N HCl was used to neutralize the 1% NaOH treated sample. After pretreatment, 300 μL of 0.1 m sodium acetate buffer pH 5.0 containing 40 μg of tetracycline, 10 mg cellulose, and 1 mg ß‐glucosidase was added. After 24, 48, and 72 h of incubation at 37 °C with shaking (180 r.p.m.), samples were centrifuged (15 000 ***g*** for 3 min), and 5 μL of the supernatant was collected for the measurement of reducing sugar using the DNS (3,5‐dinitrosalicylate) assay (Miller, 1959). The DNS reaction was performed by mixing 5 μL of the sample and 5 μL of water with 90 μL of DNS reagent in a PCR tube, followed by incubation at 95 °C for 6 min. Reducing sugar was quantified by measuring the absorbance at ƛ550. Glucose solutions were used as standards.

## Supporting information


**Figure S1** Schematic diagram of vector construction for bicistronic gene expression under the control of the DX15 promoter used in producing transgenic plants.
**Figure S2** PtrMYB221, a poplar MYB transcription factor, is the closest homolog of EgMYB1.
**Figure S3** Stem‐specific expression of PdGA20ox1 transcripts in transgenic poplar plants.
**Figure S4** Irregular xylem phenotype of transgenic Arabidopsis plants.
**Figure S5** Contents of cell wall components.
**Figure S6** Observation of over‐winter growth of transgenic poplars in LMO field.
**Table S1** Primers used in this study.
