# Supplementary material for: Wood forming tissue‐specific bicistronic expression of PdGA20ox1 and PtrMYB221 improves both the quality and quantity of woody biomass production in a hybrid poplar
Source: Plant Biotechnol J. 2018 Dec 5;17(6):1048–57. doi: 10.1111/pbi.13036 (PMC6523601; doi:10.1111/pbi.13036)
Supplement: Supplementary file 1 — Figure S1 Schematic diagram of vector construction for bicistronic gene expression under the control of the DX15 promoter used in producing transgenic plants. Figure S2 PtrMYB221, a poplar MYB transcription factor, is the closest homolog of EgMYB1. Figure S3 Stem‐specific expression of PdGA20ox1 transcripts in transgenic poplar plants. Figure S4 Irregular xylem phenotype of transgenic Arabidopsis plants. Figure S5 Contents of cell wall components. Figure S6 Observation of over‐winter growth of transgenic poplars in LMO field. Table S1 Primers used in this study. [file PBI-17-1048-s001.docx]

**SUPPORTING INFORMATION**

**Table S1. Primers used in this study**

**Figure S1. Schematic diagram of vector construction for bicistronic gene expression under the control of the DX15 promoter used in producing transgenic plants.**

(a) Schematic diagram of the DX15::PdGA20ox1-2A-PtrMYB221 construct. (b) The 2A peptide sequence with corresponding nucleotide sequence. Optimization of the nucleotide sequence was based on poplar codon usage. The PdGA20ox1 gene without the stop codon was fused to the PtrMYB221 gene as a single transcript using the 2A peptide sequence. Its mode of action is through the very inefficient peptide bonding of G and P (red) that is disrupted during translation, resulting in division into two proteins.

**Figure S2. PtrMYB221, a poplar MYB transcription factor, is the closest homolog of EgMYB1.**

(a) ClustalW alignment of the amino acid sequences of PtrMYB221/PdMYB221 (Potri.004G174400.1), PdMYB221, PtoMYB156 (AMY62793.1), Arabidopsis AtMYB4 (AT4G38620.1), and Eucalyptus EgMYB1 (Eucgr.I01406.1). The R2 and R3 MYB domains are underlined, and the EAR motif is shown in red. (b) Phylogenetic relationship. The rooted tree was prepared using a neighbor-joining method in MEGA 4.0 after alignment of the full-length amino acid sequences. The number in the branch is a bootstrap value based on 1,000 replications.

**Figure S3. Stem-specific expression of PdGA20ox1 transcripts in transgenic poplar plants.**

(a) Semi-quantitative RT-PCR using cDNA templates generated from either stem or leaf of total RNAs. PCR was performed using PdGA20ox1 primers (Table S1) with 28 cycles. The poplar *Actin2* gene was used as a loading control. (b) Quantitative RT-PCR using cDNA templates generated from stem total RNAs. The PCR primers for *PdGA20ox1* gene used in this analysis are shown in Table S1 and the reaction was performed as described in the Experimental Procedure. Error bars indicate STDEV (n=3). Sixty-day-old soil-grown poplar plants were used.

**Figure S4. Irregular xylem phenotype of transgenic Arabidopsis plants.**

Rosette level stems from 62-day-old Arabidopsis plants were used for histological analysis. Transverse stem sections of WT and five homozygous lines (#1-12 ~ #5-1) of transgenic Arabidopsis plants (i.e., DX15::PdGA20ox1-2A-PtrMYB221) were stained with 2% Phloroglucinol/HCl to visualize xylem vessel and fiber cells. Blue arrowheads indicate irregular xylem. Scale bars indicate 100 µm.

**Figure S5. Contents of cell wall components.**

Reconstructed from Table 1 of Vo et al. (2017). The chemical composition was measured by the TAPPI method (Kim et al., 2013). WT, wild type; TP, DX15::PdGA20ox1-2A-PtrMYB221 poplar.

**Figure S6. Observation of over-winter growth of transgenic poplars in LMO field.**

(a) Survival rate of WT, 35S::PdGA20ox1 and DX15::PdGA20ox1-2A-PtrMYB221 poplars after winter season. Survival rate (%) was calculated at the end of May at year 2015 from each 20 individual trees. (b) Bud flushing status of WT, 35S::PdGA20ox1 and DX15::PdGA20ox1-2A-PtrMYB221 poplars after winter season. % bud flushing was calculated by counting flushed buds from each 20 individual tree from 9^th^ April, 2015.

**Table S1.** Primers used in this study

| No | Gene ID | Gene name | Primer | Note |
| --- | --- | --- | --- | --- |
| **For gene cloning** | | | |  |
| 1 | KC461180.1 | PdGA20ox1 | Forward_aaaaagcaggctATGGGTACTTCGACTGTGAG | attB1 partial |
|  |  |  | Reverse_gacgtcacctgcaagcttaagaaggtcgaagttaagaagctg TGGCTGGTTTCTTGAGGTGA | 2A sequence |
| 2 | Potri.004G174400.1 | PtrMYB221 | Forward_cttcttaagcttgcaggtgacgtcgagtcaaacccaggtcca ATGGGAAGGTCTCCTTGCTGT | 2A sequence |
|  |  |  | Reverse_agaaagctgggtTCATTTCATCTCCAAACCTC | attB2 partial |
| 3 | attB1 |  | acaagtttgtacaaaaaagcaggct |  |
| 4 | attB2 |  | accactttgtacaagaaagctgggt |  |
| **For semi-quantitative RT-PCR** | | | |  |
| 5 | KC461180.1 | PdGA20ox1 | Forward_GCAAATCACTGGCATTTTTCCTG |  |
| 6 | Potri.004G174400.1 | PtrMYB221 | Reverse_TCATTTCATCTCCAAACCTCT |  |
| 7 | KC461180.1 | PdGA20ox1 | Forward_GTGAACAAGACAACACCTCG |  |
|  |  |  | Reverse_GCTGGTTTCTTGAGGTGAAC |  |
| 8 | AT1G49240 | AtActin8 | Forward_ATGAAGATTAAGGTCGTGGCA |  |
|  |  |  | Reverse_TCCGAGTTTGAAGAGGCTAC |  |
| 9 | Potri.019G010400.1 | PtrActin2 | Forward_GCCATCTCTCATCGGAATGGAA |  |
|  |  |  | Reverse_ AGGGCAGTGATTTCCTTGCTCA |  |
| **For quantitative real-time PCR** | | | |  |
| 10 | Potri.006G126800.1 | PtrPAL1 | Forward_TTGACTTGAGGCATTTGGAG |  |
|  |  |  | Reverse_CAATGGATAGGTAGCACTGC |  |
| 11 | Potri.019G049500.2 | Ptr4CL2 | Forward_TATTCCCAAATCGGCTTCTGG |  |
|  |  |  | Reverse_GGCAAGCTTGGCTCTCAGGTC |  |
| 12 | Potri.012G006400.2 | PtrCOAMT2 | Forward_GAGGATGCCCCATCTTATC |  |
|  |  |  | Reverse_TGCACTCAACAAGTATCACCT |  |
| 13 | Potri.001G304800.1 | PtrCCoAOMT1 | Forward_CCCTGCTCTGCCAGTTCTCGA |  |
|  |  |  | Reverse_CACCACAGATCCATTCCAC |  |
| 14 | Potri.013G157900.1 | PtrC4H1 | Forward_ACTCTGGGACGTYTGGTACA |  |
|  |  |  | Reverse_GCTTCATAGATTTACAGTGA |  |
| 15 | Potri.007G016400.1 | PtrF5H1 | Forward_TGCTATTGGGAGAGACAAGA |  |
|  |  |  | Reverse_AAACAATGAAGCAAGTGAGC |  |
| 16 | KC461180.1 | PdGA20ox1 | Forward_GTGAACAAGACAACACCTCG |  |
|  |  |  | Reverse_GCTGGTTTCTTGAGGTGAAC |  |
| 17 | Potri.019G010400.1 | PtrActin2 | Forward_GCCATCTCTCATCGGAATGGAA |  |
|  |  |  | Reverse_AGGGCAGTGATTTCCTTGCTCA |  |


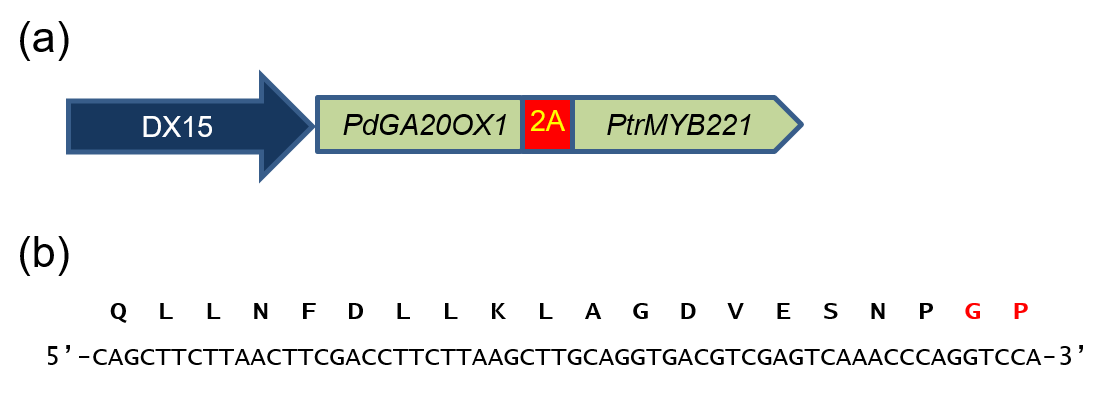


**Figure S1.**


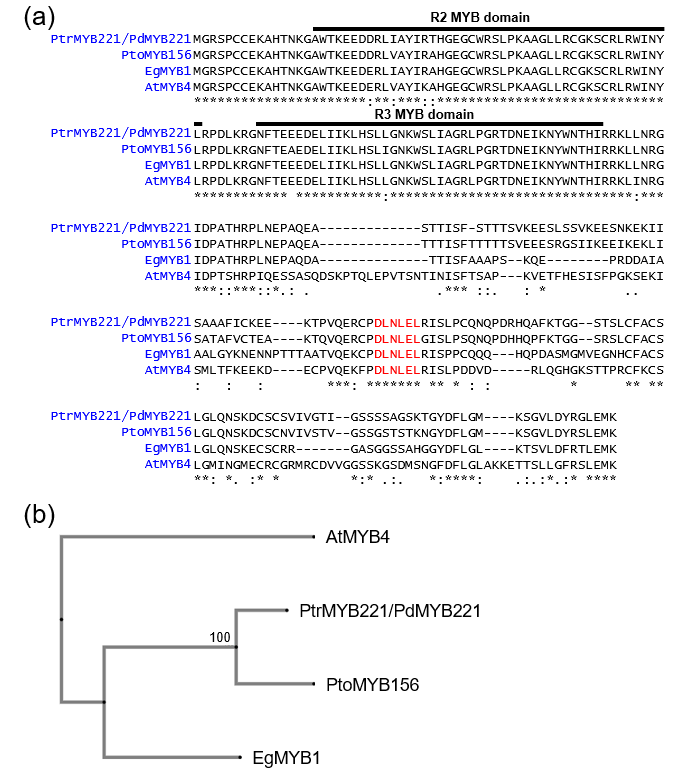


**Figure S2.**


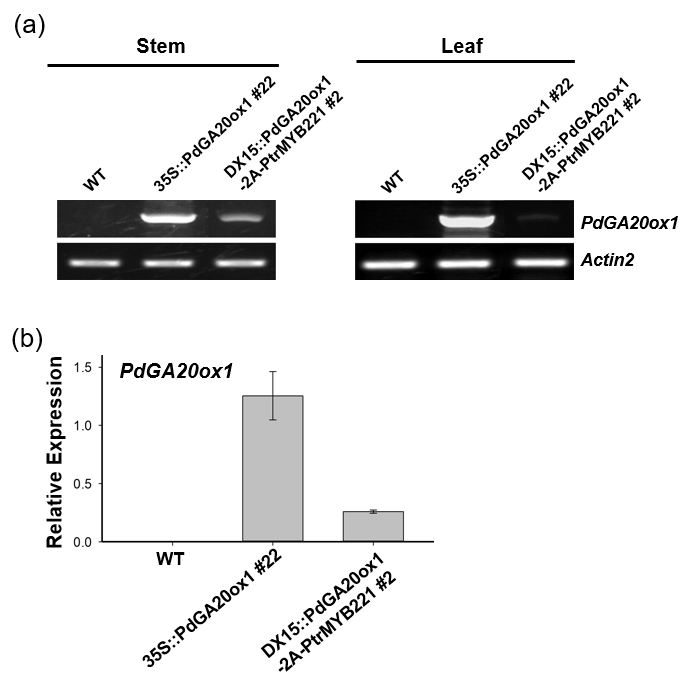


**Figure S3.**


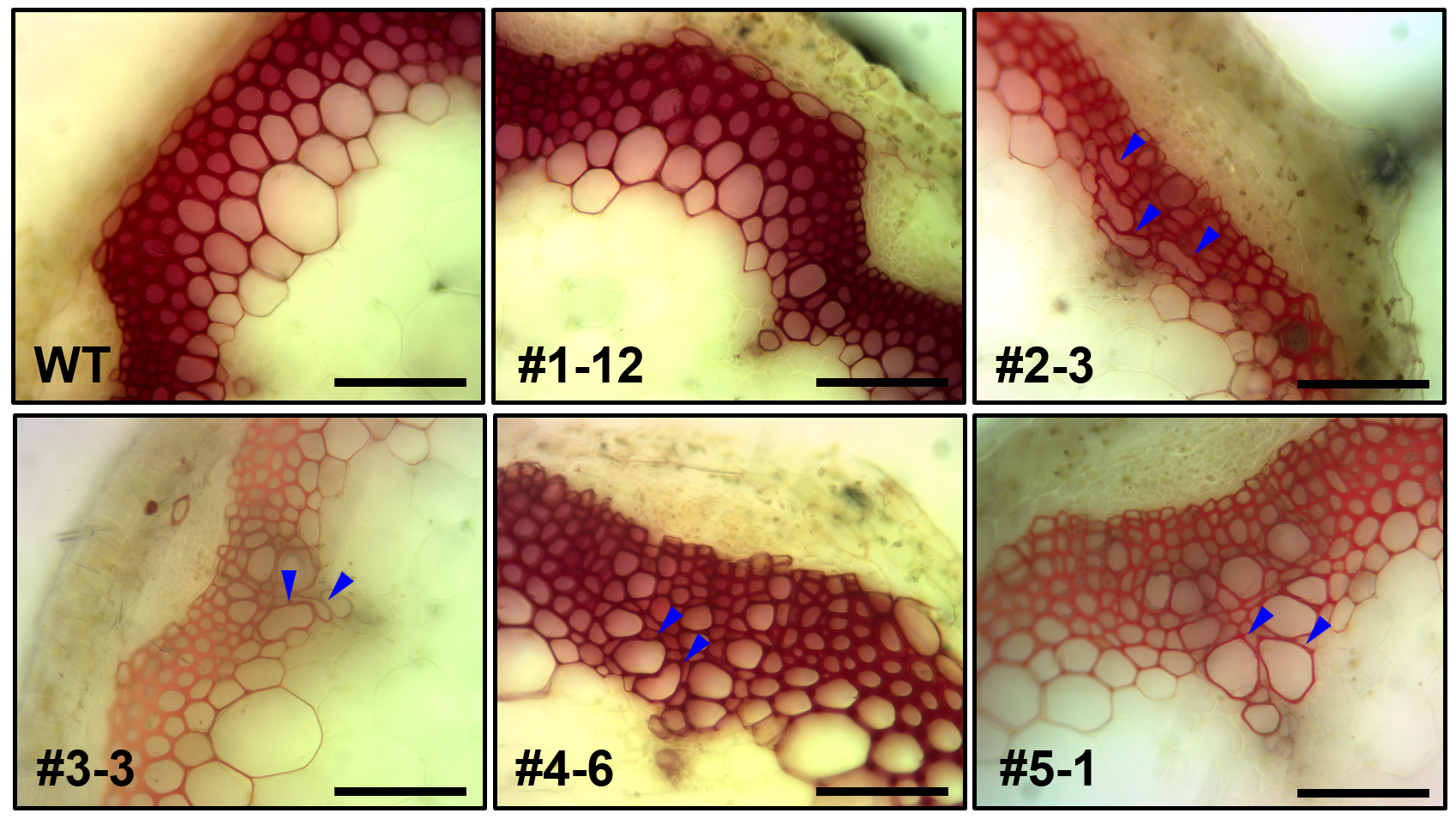


**Figure S4.**


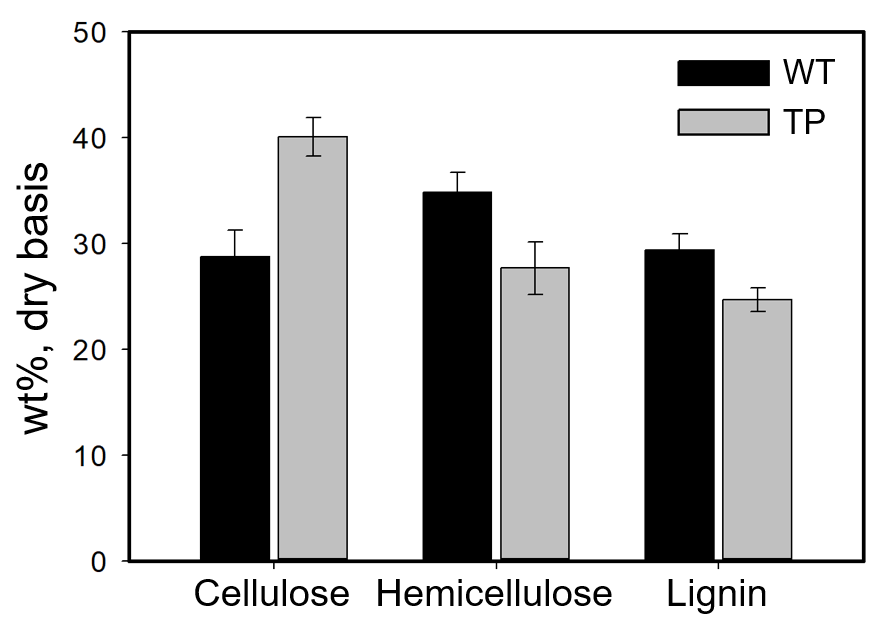


**Figure S5.**


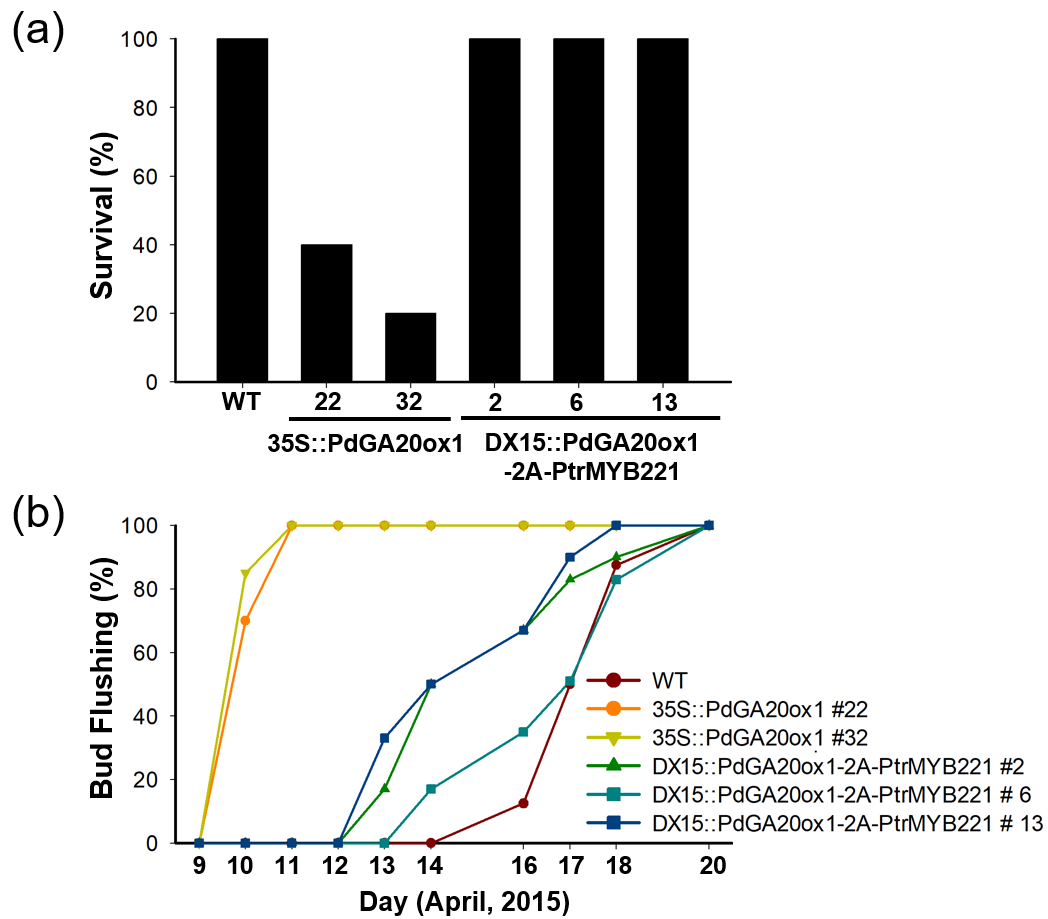


**Figure S6.**
